# Dietary Behaviour and Socioeconomic Position: The Role of Physical Activity Patterns

**DOI:** 10.1371/journal.pone.0078390

**Published:** 2013-11-06

**Authors:** Jonas D. Finger, Thorkild Tylleskär, Thomas Lampert, Gert B. M. Mensink

**Affiliations:** 1 Department of Epidemiology and Health Monitoring, Robert Koch Institute, Berlin, Germany; 2 Centre for International Health, University of Bergen, Bergen, Norway; Wageningen University, The Netherlands

## Abstract

**Background:**

The positive association between education level and health outcomes can be partly explained by dietary behaviour. We investigated the associations between education and several indices of food intake and potential influencing factors, placing special emphasis on physical-activity patterns, using a representative sample of the German adult population.

**Methods:**

The German National Health Interview and Examination Survey 1998 (GNHIES98) involved 7,124 participants aged between 18 and 79. Complete information on the exposure (education) and outcome (nutrition) variables was available for 6,767 persons. The associations between ‘education’ and indices of ‘sugar-rich food’, ‘fat-rich food’, ‘fruit-and-vegetable’ and ‘alcohol’ intake were analysed separately for men and women using multivariate logistic regression analysis. Odds ratios (OR) of education level on nutrition outcomes were calculated and adjusted for age, region (former East/West Germany), occupation, income and other influencing factors such as physical activity indicators.

**Results:**

Men and women with only a primary education had a more frequent intake of sugar-rich and fat-rich foods and a less frequent intake of fruit and vegetables and alcohol than people with a tertiary education. ‘Physical work activity’ partly explained the associations between education and sugar-rich food intake. The interference with physical work activity was stronger among men than women. No significant associations between education and energy-dense food intake were observed in the retirement-age group of persons aged 65+ and among persons with low energy expenditure.

**Conclusions:**

In Germany, adults with a low level of education report that they consume energy-dense foods more frequently – and fruit and vegetables and alcohol less frequently – than adults with a high education level. High levels of physical work activity among adults with a low education level may partly explain why they consume more energy-dense foods.

## Introduction

A healthy diet is an important factor in the prevention and treatment of non-communicable diseases [1]. In the light of the recent debate on the ‘obesity epidemic’, and also considering other health issues, consuming large amounts of low-quality, energy-dense foods or drinks (sugar-rich foods or fluids, saturated-fat-rich foods, alcohol) is regarded as unhealthy behaviour, and consuming large amounts of low-energy-dense foods (fruit and vegetables) is regarded as healthy behaviour [2]. Studies consistently show that unhealthy dietary habits cluster among people of low socioeconomic position (SEP) [3], [4], [5], [6], [7], [8], [9], [10]. However, the role of physical activity patterns should not be neglected when investigating the association between SEP and dietary behaviour. Energy balance [2] can also be achieved when high levels of energy are consumed, as long as the level of energy expenditure is also high. A previous analysis has shown that German adults with a low level of education are more physically active at work and therefore have a higher level of total energy expenditure than those with a high education level [11]. This may have an influence on specific food choices, like more frequent consumption of energy-dense foods by low-educated groups, since studies have shown that a higher energy expenditure usually goes together with a higher energy intake [12]. Studies also suggest that other factors such as body mass index, ‘self-perceived health’ and smoking status have an influence on the association between SEP and dietary behaviour [13], [14], [15] and should thus also be considered. Education level is often used as an indicator for SEP [6], [7], since it is more stable over time and the information is often more complete than that on occupation and income [16]. Studies suggest, however, that using all three SEP indicators at the same time improves our understanding of social inequalities in health behaviour [6], [17].

The aim of this analysis is to investigate the associations between ‘education’ and ‘sugar-rich food’ intake, ‘fat-rich food’ intake, ‘fruit-and-vegetable’ intake and ‘alcohol’ consumption among adults in Germany. Furthermore, it aims to examine the role of variables that may influence the association between education and dietary behaviour, such as ‘income’ level, ‘occupational’ status, ‘physical activity’, body mass index, ‘self-perceived health’ and ‘smoking’ status. The comprehensive food-frequency data from the German National Health Interview and Examination Survey 1998 (GNHIES98) enabled us to investigate these associations.

## Methods

### Study design and participants

The GNHIES98 is a cross-sectional, nationally representative health survey that was conducted in Germany between October 1997 and March 1999. The sample was selected using a multi-stage, clustered, random sampling procedure. In the first stage, 130 communities were randomly selected and stratified according to region (Federal Land) and community size to represent the structure of the Federal Republic of Germany. Adults aged between 18 and 79 were randomly selected from population registries within these communities, stratified according to 5-year age groups and gender. The net sample included 7,124 participants, which corresponds to an overall response rate of 61.4%. The cluster-sampling procedure is described in detail elsewhere [18]. The GNHIES98 was a general health survey meant to serve many data analyses. The data analysis presented in this paper is not a separate study, since the participants were not contacted again. The survey was approved by the Board of the Federal Commissioner for Data Protection Berlin, Germany [19]. Each participant gave his or her informed written consent before enrolment in the survey. All participants were informed about the study's objectives, completed a self-administered health questionnaire and underwent a physical examination. Body height and weight was measured with calibrated instruments in a standardized way. Complete information on education level (exposure) and nutrition indices (outcomes) was available for 6,767 respondents.

The response analysis revealed that the responders were more likely to report a high level of education and better self-perceived health than the non-responders [11].

### Definitions of variables

#### Nutrition outcomes

The general health questionnaire included questions on the frequency of consumption of several food groups. Information on consumption was obtained by asking the questions: ‘During the last 12 months, how often have you eaten the following foods?’, and ‘How often have you consumed the following drinks?’ The available answer categories were re-coded to obtain frequencies on a weekly basis: ‘several times a day’ (14), ‘daily or almost daily’ (7), ‘several times a week’ (2.5), ‘about once a week’ (1), ‘2–3 times a month’ (0.6), ‘once or less a month’ (0.25), and ‘almost never’ (0). The weekly frequencies of the sub-items were cumulated to sum scores of the respective food categories. Four nutrition indices were constructed by cumulating the frequencies of intake of the following food or beverage items:

*‘Sugar-rich food index’*: ‘cakes, biscuits, pastries’, ‘confectionery (e.g. sweets, pralines, chocolate)’ and ‘soft drinks’ (lemonade, fruit drinks, pop, cola, tonic water)’.

*‘Fat-rich food index’*: ‘convenience food (TV dinners)’, ‘fried or deep-fried potatoes’ and ‘fried sausage, curry sausage, hamburgers, kebabs, pizza’.

*‘Fruit and vegetable index’*: ‘green salad, raw vegetable salad or raw vegetables’, ’fresh or frozen vegetables (cooked)’ and ‘fresh fruits’.

*‘Alcohol consumption index’*: ‘wine, champagne or fruit wine’, ‘beer’ and ‘spirits’. The alcohol consumption index was constructed in terms of grams of alcohol consumed per day by assigning beverage-specific, alcohol-content weights to the respective standard units of drinks, multiplying them by the respective frequency categories and cumulating the sub-item quantities to a sum score.

This short instrument only produces rough estimates on dietary behaviour and was used to rank individuals – instead of using the continuous food-frequency scores for analysis. Quintiles were calculated for all indices, and the population was divided into 40% versus 60% using the upper limit of the 3^rd^ quintile of the high energy-dense food indices (sugar, fat, alcohol) to define frequency of intake as ‘high’. For the fruit-and-vegetable index, the lower limit of the 3^rd^ quintile was used to define frequency of intake as ‘low’. The following cut-points were used. ‘*Sugar-rich food intake*’: 7.5 times per week for men, 5.25 for women; ‘*fat-rich food intake*’: 2.0 for men and 1.25 for women; ‘*fruit-and-vegetable intake*’: 7.5 times per week for men and 10.5 for women; and ‘*alcohol intake*’: 14.3 grams per day for men and 2.1 for women.

#### Socioeconomic position (SEP)

*‘Education’* was assessed using two questions on the highest school-leaving certificate and the highest vocational-training certificate achieved by the respondent. A categorical education variable (primary, secondary, tertiary education) was generated by applying the ‘Comparative Analysis of Social Mobility in Industrial Nations’ (CASMIN) approach adapted to the German education system [20].

*‘Income’* was constructed based on two questions asking about the ‘household's approximate net income’ and the ‘household size’ by applying household weights recommended by OECD [21]. The income index was used to construct a categorical variable dividing the population into three equal groups (tertiles) in order to define income as ‘low’, ‘middle’ or ‘high’.

*‘Occupation’* was constructed based on one question asking about the current or last professional position. The ‘Occupational Prestige in Comparative Perspective’ approach for Germany was applied to categorize respondents into three categories of occupational status (low, middle, high) [22].

#### Covariates

Four physical activity covariates were constructed based on the following questions:

*‘Vigorous work activity’*: ‘Is your present occupation characterized by vigorous physical activity? Yes/No’.

*‘Sports activity’*: ‘How often do you engage in sports: regularly, more than 4 hours per week; regularly, 2–4 hours per week; regularly, 1–2 hours per week; less than 1 hour per week; no sports activity?’

*‘Total energy expenditure’*: ‘How much time per day (24 hours) do you spend on average doing the following: a) Sleeping, relaxing; b) Sitting down; c) Light activities; d) Moderately vigorous activities; e) Vigorous activities?’ A 24-hour total-energy expenditure index was constructed by assigning metabolic equivalent (MET) values [23] to the respective activity categories (sleeping = 0.9, sitting = 1.3, light activity = 2.5, moderately vigorous activity = 4.5, vigorous activity = 6 MET) and cumulating activities over 24 hours. The activity information was assessed separately for weekdays and weekend days. Quintiles were calculated for the energy expenditure index, and the population was categorized into five equal groups.

*‘Sitting time weekdays’* was calculated using the item on time spent sitting on weekdays. Again, the population was categorized into five equal groups by calculating quintiles.

*Body mass index* (BMI) was calculated on the basis of physical examination data as body weight (kg)/height (m)^2^, and the population was divided into three categories of BMI according to the guidelines of the World Health Organization (BMI<25, 25–<30, and ≥30 kg/m^2^). Height was measured without shoes, and weight was measured in light clothes.

*‘Self-perceived health’* was assessed by asking the question: ‘In general, would you say your health is: excellent; very good; good; fair; poor?’

*‘Smoking status’* was assessed in three categories: ‘current smoker’, ‘past smoker’ and ‘never smoked’.

‘*Region*’ was defined as former East versus former West Germany.

### Statistical analysis

The statistical analyses were performed using the software package STATA SE 12.0. In all statistical analyses, the cluster structure of the multi-stage sample was accounted for by using survey-design procedures. These procedures lead to wider confidence intervals compared to standard statistical procedures, which assume simple random sampling. Simple bivariate analyses were performed using logistic regression analyses. Possible influencing factors for the investigated associations were selected in a first step on the basis of knowledge and theories from literature; inference statistics were then used to clarify their statistical significance. Confounding and interaction of covariates on the association between education level and nutrition variables were examined by performing stepwise logistic regression analyses (*Model 1*: outcome and exposure variable; *Model 2*: Model 1+covariate; *Model 3*: Model 2+interaction term of exposure*covariate). Estimations of each model were stored at each stage and tested for model fit using a likelihood-ratio test (lrtest) by comparing the post-estimations of the respective models. Confounding of a covariate was given if the lrtest was significant comparing the post-estimations of Model 1 and Model 2. Interaction was given if, in addition, the lrtest was significant comparing Model 2 and Model 3. The age- and region-adjusted associations between education and nutrition variables in the basic models were subsequently adjusted for occupation, income and other significant confounders for the respective associations. When adjusting for covariates, we used the age strata 18–39, 40–59 and 60–79; the BMI categories <25, 25–<30, and ≥30 kg/m^2^; the ‘self-perceived health’ strata ‘excellent’, ‘very good’, ‘good’, ‘fair’ and ‘poor’; the ‘work activity’ strata ‘vigorous work activity’ and ‘no vigorous work activity’; the ‘sports activity’ strata ‘no sports activity’, <1, 1–2, 2–4 and >4 hours per week; quintiles of the ‘total energy expenditure’, ‘sitting time weekdays’, ‘sugar-rich food’, ‘fat-rich food’, ‘fruit and vegetable’ and ‘alcohol’ index; and the ‘smoking status’ categories ‘current smoker’, ‘past smoker’ and ‘never smoked’. Missing values of the covariates were included in the statistical analyses by generating a separate category for missing values (the numbers are shown in Table 1). Furthermore, subgroup analyses were performed, stratifying by identified effect-modifying variables. Finally, the Baron and Kenny [24] statistical mediation criteria were tested for hypothesized mediating factors: Given a significant relation between the independent and dependent variable and a significant relation between the independent variable and the hypothesized mediating variable, the mediating variable is significantly related to the dependent variable when both the independent and the mediating variable are in the model; the coefficient relating the independent variable to the dependent variable must be larger in absolute terms than the coefficient relating the independent variable to the dependent variable in the regression model, which includes both the independent variable and the mediating variable [24], [25].

**Table 1:** Selected variables of participants aged 18–79 in relation to key outcome variables.

	Study sample		Sugar-rich food intake mean (times/week)	Fat-rich food intake mean (times/week)	Fruit-and-vegetable intake mean (times/week)	Alcohol intake mean (grams/day)
	n	%				
**Total sample**	6767		6.7	1.9	12.1	9.6
**Age group (years)**						
17–39	2716	40	8.8	2.4	11.0	9.2
40–59	2553	38	5.7	1.6	12.7	11.1
60–79	1498	22	4.7	1.4	13.1	7.9
**Gender**						
men	3298	49	7.3	2.3	10.5	15.5
women	3469	51	6.3	1.5	13.7	4.0
**Region in Germany**						
former East	2304	34	6.7	1.7	12.9	9.9
former West	4463	66	6.8	1.9	11.7	9.5
**Educational level**						
primary	2901	43	6.4	1.9	12.1	8.4
secondary	2917	43	7.5	2.0	12.0	9.4
tertiary	949	14	5.7	1.6	12.7	14.0
**Occupational status**						
low	2658	39	6.7	1.9	11.9	8.4
middle	2214	33	6.4	1.7	12.6	9.2
high	1258	19	6.2	1.7	11.9	14.2
missing	637	9	9.1	2.7	11.8	7.4
**Income level**						
low	1814	25	7.2	2.0	12.0	9.0
middle	1841	26	6.7	1.8	12.1	9.6
high	1793	24	6.5	1.8	11.6	12.2
missing	1319	17	6.4	1.9	13.1	7.0
**Body mass index (kg/m^2^)**						
<25	2711	40	7.7	2.1	12.0	8.6
25–<30	2630	39	6.4	1.8	12.0	11.0
≥30	1383	20	5.6	1.6	12.6	9.1
missing	43	<1				
**Self-perceived health**						
excell./very good	1345	20	7.8	2.1	12.0	10.0
good	4233	63	6.7	1.8	12.1	9.8
fair/poor	1186	18	5.8	1.8	12.2	8.6
missing	3	<1				

## Results

### Participants

The description of participants in relation to selected variables and nutrition outcome variables is presented in Table 1.

Table 2 shows the crude associations (odds ratios) between the outcome variables according to the exposure variable (education) and other covariates used in the multivariate models. A higher level of vigorous work activity and total energy expenditure was associated with a higher frequency of sugar-rich and fat-rich food intake. A lower level of sitting time weekdays was associated with a higher frequency of fruit-and-vegetable intake.

**Table 2:** Crude odds ratios (OR) of nutrition indicators a according to selected key variables, adults aged 18–79.

	No. in sample	High sugar-rich food intake	High fat-rich food intake	Low fruit-and-vegetable intake	High alcohol intake
		OR 95% CI	OR 95% CI	OR 95% CI	OR 95% CI
**Total**	6767				
**Education**					
primary	2901	1.3 (1.0–1.5)	1.0 (0.9–1.2)	1.3 (1.1–1.6)	1.0
secondary	2917	1.6 (1.4–1.9)	1.4 (1.2–1.7)	1.4 (1.2–1.7)	1.3 (1.2–1.5)
tertiary	949	1.0	1.0	1.0	2.5 (2.2–2.9)
**Occupation**					
low	2658	1.2 (1.0–1.4)	1.2 (1.0–1.4	1.2 (1.0–1.3)	1.0
middle	2214	1.0 (0.9–1.2)	1.0 (0.8–1.1)	0.8 (0.7–1.0)	1.3 (1.1–1.5)
high	1258	1.0	1.0	1.0	2.3 (2.0–2.7)
**Income**					
low	1814	1.2 (1.1–1.4)	1.1 (1.0–1.3	1.0 (0.9–1.2)	1.0
middle	1841	1.1 (1.0–1.3)	0.9 (0.8–1.1)	0.9 (0.8–1.0)	1.3 (1.1–1.5)
high	1793	1.0	1.0	1.0	1.9 (1.6–2.2)
**Vigorous work activity**					
yes	1522	1.5 (1.3–1.7)	1.2 (1.1–1.4)	1.2 (1.1–1.4)	0.9 (0.8–1.0)
no	2289	1.0	1.0	1.0	1.0
**Sports activity ≥2 hours/week**					
yes	1254	1.1 (0.9–1.2)	1.2 (1.1–1.4)	1.2 (1.0–1.4)	1.4 (1.2–1.6)
no	5498	1.0	1.0	1.0	1.0
**Sitting time weekdays (hours)**					
≥8	2113	0.9 (0.8–1.1)	1.1 (1.0–1.3)	1.3 (1.1–1.4)	1.2 (1.0–1.3)
5–<8	1905	0.9 (0.8–1.0)	0.9 (0.8.1.0)	1.1 (0.9–1.2)	0.9 (0.8–1.1)
<5	2730	1.0	1.0	1.0	1.0
**Total energy expenditure**					
low	2189	1.0	1.0	1.0	1.0
middle	2233	1.0 (0.8–1.1)	0.9 (0.8–1.1)	0.9 (0.8–1.0)	0.8 (0.7–1.0)
high	2286	1.3 (1.1–1.4)	1.3 (1.1–1.4)	1.0 (0.9–1.2)	1.4 (1.2–1.7)
**Body mass index (kg/m^2^)**					
<25	2711	1.8 (1.5–2.0)	1.6 (1.4–1.9)	1.2 (1.0–1.3)	1.1 (0.9–1.2)
25–<30	2630	1.3 (1.1–1.5)	1.3 (1.1–1.5)	1.1 (0.9–1.2)	1.4 (1.2–1.6)
≥30	1383	1.0	1.0	1.0	1.0
**Self-perceived health**					
excell./very good	1345	1.7 (1.5–2.0)	1.7 (1.4–1.9)	1.0 (0.9–1.2)	1.7 (1.4–2.0)
good	4233	1.2 (1.1–1.4)	1.3 (1.1–1.4)	1.0 (0.9–1.1)	1.4 (1.2–1.6)
fair/poor	1186	1.0	1.0	1.0	1.0

a Fruit-and-vegetable, sugar-rich food, fat-rich food and alcohol intake is defined as ‘high’ using the upper limit of the 3^rd^ quintile as the cut-point dividing the population in 40% versus 60%.

### Multivariate analyses

#### Sugar-rich food intake

Work activity (only among men), sports activity (only among women), BMI, smoking status (only among women), self-perceived health (only among women), fat-rich food intake, fruit-and-vegetable intake (only among women) and alcohol intake were significant confounders (95% level of confidence) for the association between education and high sugar-rich food intake (Table 3). After adjustment for these variables, a significant negative association between education and sugar-rich food intake remained among women.

**Table 3:** Stepwise adjusted odds ratios (OR) of sugar-rich and fat-rich food intake according to education, men and women aged 18–79.

	High sugar-rich food intake		High fat-rich food intake	
	Model 1^a^OR 95%CI	Final Model^b^OR 95%CI	Model 1^a^OR 95%CI	Final Model^c^OR 95%CI
**Men (n = 3298)**				
**Education**				
primary	1.4 (1.1–1.8)*	1.3 (1.0–1.7)	1.3 (1.1–1.7)*	1.2 (0.9–1.6)
secondary	1.3 (1.0–1.6)	1.2 (0.9–1.6)	1.3 (1.0–1.7)*	1.2 (1.0–1.6)
tertiary	1.0	1.0	1.0	1.0
**Occupation**				
low		0.8 (0.7–1.0)		0.9 (0.7–1.1)
middle		0.8 (0.7–1.0)		0.9 (0.8–1.2)
high		1.0		1.0
**Income**				
low		1.1 (0.9–1.3)		1.2 (0.9–1.5)
middle		1.1 (0.9–1.4)		1.0 (0.8–1.2)
high		1.0		1.0
**Women (n = 3469)**				
**Education**				
primary	1.6 (1.2–2.1)*	1.4 (1.0–2.0)	1.5 (1.1–2.0)*	1.2 (0.8–1.6)
secondary	1.5 (1.2–2.0)*	1.4 (1.0–1.9)*	1.3 (1.0–1.7)	1.1 (0.8–1.5)
tertiary	1.0	1.0	1.0	1.0
**Occupation**				
low		1.1 (0.8–1.4)		0.8 (0.6–1.0)
middle		1.0 (0.8–1.3)		0.9 (0.7–1.0)
high		1.0		1.0
**Income**				
low		1.0 (0.8–1.2)		0.8 (0.7–1.1)
middle		1.0 (0.8–1.2)		1.0 (0.8–1.2)
high		1.0		1.0

a Model adjusted for age groups and regional strata east vs. west Germany.

b Adjusted as Model 1 and also for vigorous work activity (among men), sports activity (among women), BMI, smoking status (among men), self-perceived health (among women), fat-rich food intake, fruit-and-vegetable intake (among women) and alcohol intake.

c Adjusted as Model 1 and also for sports activity (among men), sitting time weekdays (among women), BMI, self-perceived health (among men), smoking status, sugar-rich food intake and fruit-and-vegetable intake.

* Significant on a 95% level of confidence.

#### Fat-rich food intake

Sports activity (only among men), sitting time weekdays (only among women), BMI, self-perceived health (only among men), smoking status, sugar-rich food intake and fruit-and-vegetable intake were significant confounders for the association between education and fat-rich food intake (Table 3). No significant association remained after adjustment.

#### Fruit-and-vegetable intake

Sports activity, sitting time weekdays (only among men), total energy expenditure (only among women), BMI, smoking status, sugar-rich food intake, fat-rich food intake and alcohol intake were significant confounders for the association between education and fruit-and-vegetable intake (Table 4). After adjustment, a significant negative association between education and fruit-and-vegetable intake and between occupation and fruit-and-vegetable intake remained among women.

**Table 4:** Stepwise adjusted odds ratios (OR) of fruit-and-vegetable and alcohol intake according to education, men and women aged 18–79.

	Low fruit-and-vegetable intake		High alcohol intake	
	Model 1^a^OR 95%CI	Final Model^b^OR 95%CI	Model 1^a^OR 95%CI	Final Model^c^OR 95%CI
**Men (n = 3298)**				
**Education**				
primary	1.4 (1.1–1.8)*	1.3 (0.9–1.6)	1.0	1.0
secondary	1.3 (1.1–1.6)*	1.2 (1.0–1.6)	1.2 (1.0–1.5)*	1.2 (1.0–1.5)*
tertiary	1.0	1.0	1.6 (1.3–1.9)*	1.6 (1.2–2.0)*
**Occupation**				
low		1.2 (0.9–1.4)		1.0
middle		1.1 (0.9–1.3)		1.2 (0.9–1.5)
high		1.0		1.2 (0.9–1.5)
**Income**				
low		1.0 (0.8–1.3)		1.0
middle		0.8 (0.7–1.0)		1.0 (0.8–1.3)
high		1.0		1.1 (0.9–1.4)
**Women (n = 3469)**				
**Education**				
primary	1.8 (1.4–2.3)*	1.3 (1.0–1.9)	1.0	1.0
secondary	1.5 (1.2–2.0)*	1.4 (1.1–1.8)*	1.6 (1.3–1.9)*	1.3 (1.1–1.5)*
tertiary	1.0	1.0	2.6 (2.0–3.3)*	1.6 (1.2–2.1)*
**Occupation**				
low		1.4 (1.0–1.8)*		1.0
middle		1.0 (0.8–1.3)		1.4 (1.2–1.7)*
high		1.0		1.5 (1.1–1.9)*
**Income**				
low		0.9 (0.7–1.1)		1.0
middle		1.0 (0.8–1.2)		1.4 (1.1–1.6)*
high		1.0		1.4 (1.1–1.7)*

a Model adjusted for age groups and regional strata east vs. west Germany.

b Adjusted as Model 1 and also for sports activity, sitting time weekdays (among men), total energy expenditure (among women), BMI, smoking status, sugar-rich food intake, fat-rich food intake and alcohol intake.

c Adjusted as Model 1 and also for sitting time weekdays (among men), vigorous work activity (among women), total energy expenditure (among men), self-perceived health, smoking status, sugar-rich food intake, fat-rich food intake and fruit-and-vegetable intake (among men).

* Significant on a 95% level of confidence.

#### Alcohol intake

Sitting time weekdays (only among men), vigorous work activity (only among women), total energy expenditure (only among men), self-perceived health, smoking status, sugar-rich food intake, and fruit-and-vegetable intake (only among men) were significant confounders for the association between education and alcohol intake (Table 4). After adjustment, significant positive associations remained between all SEP variables (education, occupation, income) and alcohol intake among women, and a significant positive association between education and alcohol intake remained among men.

### Other analyses

Total energy expenditure and age were significant effect modifiers for the associations between education and sugar-rich food intake and between education and fat-rich food intake. Both associations were stronger and significant among persons with high total energy expenditure as well as among working-age respondents (18–64 years). No significant associations were observed in the stratum of persons with low total energy expenditure or among persons aged 65+ (Table 5). Within the stratum of persons with high energy expenditure, sports activity ≥2 hours was reported by 17.6% (95% CI, 15.3–20.0) of persons with primary education, 23.8% (21.1–26.3) of persons with secondary education, and 34.0% (27.4–40.6) of persons with tertiary education. The corresponding percentages for vigorous work activity were 72.6% (69.2–76.0), 65.5% (62.2–68.9) and 30.7% (22.8–38.5), respectively. And the corresponding mean MET values of the 24-hour total energy expenditure index were 62.1 MET/24 hours (61.6–62.6), 62.3 MET/24 hours (61.8–62.8) and 60.0 MET/24 hours (59.0–61.0), respectively.

**Table 5:** Odds ratios (OR) of the association between education and nutrition indicators a stratified by age and total energy expenditure, adults aged 18–79.

	No. in sample	High sugar-rich food intake	High fat-rich food intake
		OR 95% CI	OR 95% CI
**Stratified analysis by energy expenditure** b			
**low**	2189		
Primary education	920	1.3 (0.9–1.7)	1.0 (0.8–1.3)
Secondary education	854	1.0 (0.7–1.4)	1.0 (0.7–1.2)
Tertiary education	415	1.0	1.0
**middle**	2233		
Primary education	930	1.3 (0.9–1.8)	1.1 (0.8–1.5)
Secondary education	978	1.3 (0.9–1.7)	1.0 (0.7–1.3
Tertiary education	325	1.0	1.0
**high**	2286		
Primary education	1023	2.1 (1.5–2.9)	1.5 (1.1–2.2)
Secondary education	1062	1.7 (1.2–2.4)	1.3 (0.9–2.0)
Tertiary education	201	1.0	1.0
**Stratified analysis by age group** c			
**<65**	5839		
Primary education	2222	1.4 (1.2–1.8)	1.1 (0.9–1.3)
Secondary education	2757	1.6 (1.4–2.0)	1.4 (1.2–1.7)
Tertiary education	860	1.0	1.0
**65+**	928		
Primary education	679	1.2 (0.7–2.1)	0.9 (0.6–1.5)
Secondary education	160	0.9 (0.6–1.5)	1.1 (0.6–2.0)
Tertiary education	89	1.0	1.0

a Sugar-rich and fat-rich food intake is defined as ‘high’ using the upper limit of the 3^rd^ quintile as the cut-point dividing the population in 40% versus 60%.

b Models adjusted for age groups and regional strata east vs. west Germany.

c Models adjusted for regional strata east vs. west Germany.

Vigorous work activity fulfilled the criteria of mediation for the association between education and sugar-rich food intake among men (95% level of confidence) and women (90% level of confidence). The inclusion of work activity in the age- and region-adjusted model of the association of education and sugar-rich food intake, Table 6, explained 36% of the association between education and sugar-rich food intake among men and 18.6% among women when comparing persons with tertiary and primary education. The corresponding percentages were 36% among men and 12% among women when comparing persons with tertiary and secondary education.

**Table 6:** Odds ratios (OR) of the association between education and high sugar-rich food intake a adjusted for vigorous work activity b, men and women aged 18–79.

	Study sample		Basic Model; age+region+education		Model 1; Basic Model+vigorous work activity	
	n	%	OR 95% CI	P-value	OR 95% CI	P-value
**Men**						
**Education**						
primary	1381	36	1.41 (1.11–1.80)	0.006	1.26 (0.99–1.61)	0.063
secondary	1313	42	1.25 (1.00–1.58)	0.055	1.17 (0.93–1.48)	0.187
tertiary	604	29	1.0		1.0	
**Vigorous work activity**						
Yes	925	28			1.0	
No	1206	37			0.72 (0.61–0.85)	0.000
Missing	1165	35			0.75 (0.60–0.94)	0.013
**Women**						
**Education**						
primary	1520	34	1.59 (1.20–2.12)	0.002	1.48 (1.10–2.00)	0.011
secondary	1604	45	1.51 (1.16–1.97)	0.002	1.45 (1.10–1.90)	0.009
tertiary	345	32	1.0		1.0	
**Vigorous work activity**						
Yes	607	18			1.0	
No	1088	31			0.81 (0.64–1.02)	0.080
Missing	1762	51			0.96 (0.76–1.22)	0.750

a Sugar-rich food intake is defined as ‘high’ using the upper limit of the 3^rd^ quintile as the cut-point dividing the population in 40% versus 60%.

b Categories used for adjustment: ‘vigorous work activity’, ‘no vigorous work activity’, ‘missing’.

## Discussion

In this nationwide, cross-sectional study of a randomly-selected sample of adults in Germany it is observed that, crudely adjusted for age and region, adults with a low level of education consume sugar- and fat-rich foods more often, and fruit and vegetables and alcohol less often than adults with a high education level. These observations are in line with the findings of other studies [7], [26], [27]. These associations become smaller or disappear when controlling for physical activity variables and other influencing factors. Education shows a stronger independent association with the nutrition variables than occupation and income; this also confirms the findings of other studies [3], [5], [6], [28].

We observed that the association between level of education and sugar-rich food intake is partially mediated by physical work activity. This finding supports to some extent the initial hypothesis that low-educated persons may consume energy-dense food more often because they have a greater demand for energy due to their physically-demanding job. In line with our study findings, Lallukka et al. found that occupation status mediated the association between education and healthy dietary habits [5]. The assumption that work-related physical-activity patterns determine the higher frequency of energy-dense food intake among low-education groups is also supported by the finding that education differences in energy-dense food intake were only observed in the working-age stratum (<65 years of age), but not in the retirement-age stratum (65+ years), where SEP disparities in work-related activity patterns tend to disappear. The interference of physical work activity is stronger among men than women. One reason could be that men are more likely to have physically-demanding jobs than women [11], [29], [30] and that this factor therefore plays a more important role among men than women. One limitation of this study is that the physical-activity domain ‘household/care-giving activity’ was not measured separately. Studies suggest that such activities are more common among women [29]. Assuming that there is a negative association between education and household physical activity level [31], it could be that this factor, not considered in our study, explains part of the remaining association between education and sugar-rich food intake among women.

The initial hypothesis that the level of total energy expenditure may also mediate the association between education and high energy-dense food intake is not confirmed. Total energy expenditure is found to be an effect modifier (moderator) for the association between education and sugar-rich and fat-rich food intake. Subgroup analyses show that education disparities on sugar- and fat-rich food intake are only observed in the stratum of individuals with high total energy expenditure. The possible variance in nutrition is greater if there is high energy expenditure. Thus, it may be that educational differences in unhealthy energy-dense food intake only appear if there is a need to compensate high energy expenditure. High-education groups with high energy expenditure perform more sports activity, whereas low-education groups with high energy expenditure perform more physical work [11]. One study shows that the type of physical activity may influence appetite control, in terms that ‘vigorous-intensity aerobic activity’, which often correlates with sports activity, produces a counterintuitive reduction in hunger, leading to a negative energy balance in the short term [32]. Furthermore, persons who engage voluntarily in health-enhancing exercise in their leisure time may also be more health-conscious in other fields such as nutrition [33]. Regular exercisers (mainly high-educated) may compensate their high demand of energy by consuming more energy from other sources than sugar or fat (e.g. whole-grain products), compared to physical workers (mainly low-educated) who are obliged by contract to be active at work. Furthermore, the level of energy expenditure within the stratum of high energy expenditure is not homogenous and differs according to education. People with a primary and secondary education have higher energy expenditure than those with a tertiary education. This may be another explanation for the higher energy-dense food intake among persons with lower education in this stratum.

**Figure 1:**
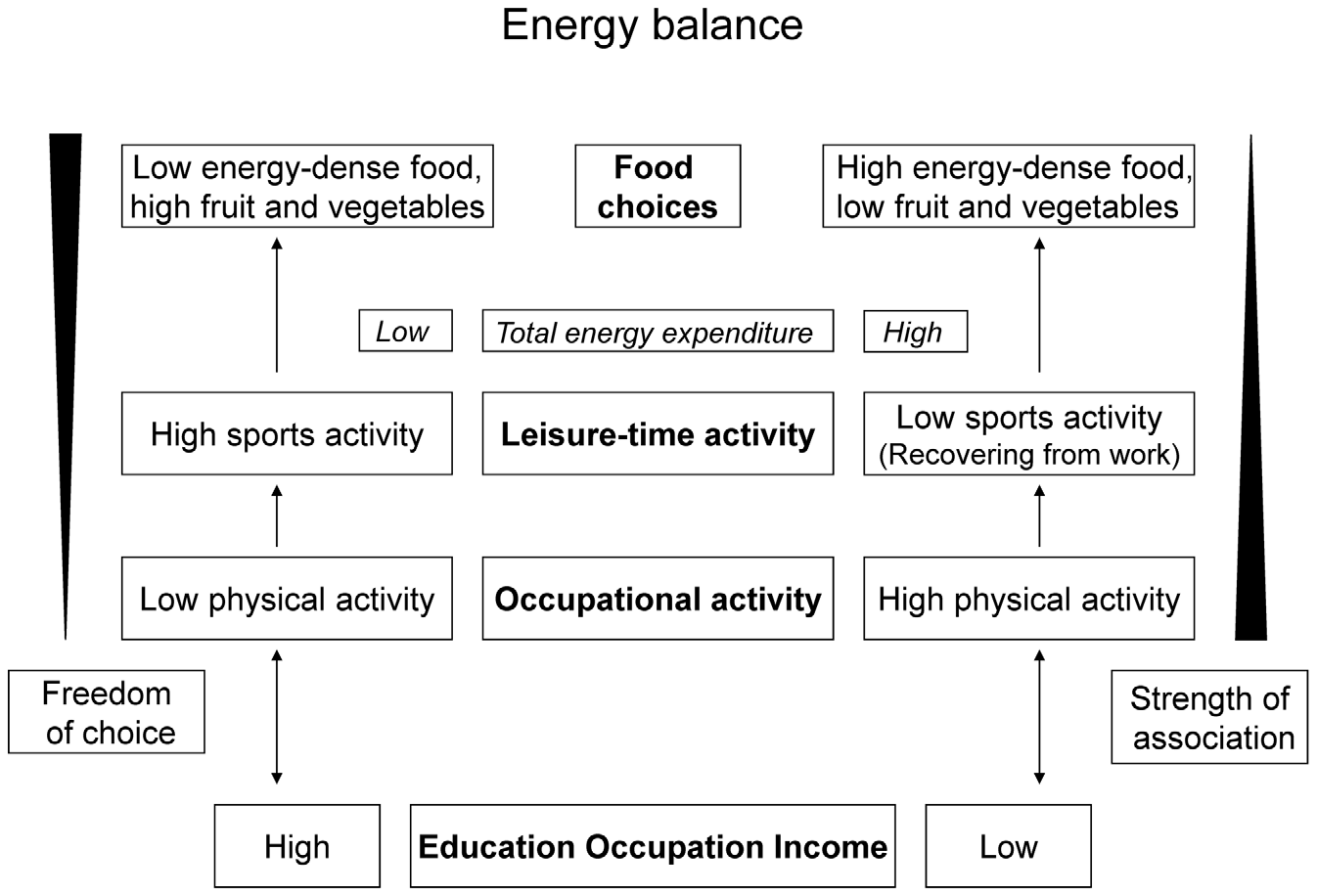
Pathways of physical activity and dietary behaviour according to socioeconomic position (SEP).

Potential pathways on how SEP may influence physical-activity and diet-related behaviours are depicted in Figure 1. SEP may influence the occupational physical-activity level in such a way that low-status groups are more likely to have physically-demanding jobs and high-status groups sedentary jobs [11]. The association between SEP and occupational physical activity may be particularly strong because work-related activity is determined by contract and the individual has limited behavioural control to change working tasks (non-volitional behaviour [34]). The level of physical work activity may in return influence leisure-time physical-activity behaviour, in that people who are sedentary at work are more active in leisure time than those who are physically active at work [11]. Since leisure-time physical activity is not determined by contract, behavioural control is greater and the association with SEP perhaps weaker. Total energy expenditure is the sum of leisure-time and occupational activity. Occupational activity usually corresponds to an 8-hour working day and leisure-time activity to shorter periods [35]; the occupational activity level therefore dominates the level of total energy expenditure. Thus, physical workers (mainly low-SEP groups) have a higher level of total energy expenditure than sedentary workers (mainly high-SEP groups), although they are less active in leisure time [11]. As a result, low-SEP groups may consume more energy-dense foods than high-SEP groups to reach energy balance. The associations between dietary behaviours and SEP may be weaker than that between physical-activity behaviour and SEP because food choices are not directly determined by contract, although they are influenced by the level of total energy expenditure. Hence, the clustering of ‘unhealthy’ behaviours among low-status groups, in terms of low leisure-time activity and high energy-dense food intake (sugar, fat) [10], may be explained in part by the structural, underlying factor of physically-demanding work. In addition to structural factors, however, awareness of diet and health, both as better knowledge and as a more positive attitude among the higher educated, may also have contributed to a more healthy dietary behaviour in this group [36]. Moreover, the higher intake of energy through the higher consumption of alcohol among the higher compared to the lower educated, may also be a reason why they consume less energy from other sources such as sugar or fat.

Although the magnitudes of the SEP gradients on dietary behaviour may have changed since 1998, we assume that the results are still relevant, since trend studies have shown that the association between socioeconomic position and dietary habits has been quite stable over time [37], [38].

### Limitations

No causal inference can be drawn when interpreting these results, since the study relies on cross-sectional data, and dietary behaviour and physical activity were assessed on the basis of self-reports.

The GNHIES98 is a general health survey. Although a nutrition survey was conducted in a subsample, it was not feasible to assess dietary behaviour and physical activity for the entire sample in a comprehensive way. Hence, only a brief food-frequency questionnaire was used covering the main food items, but certainly not all. The food-frequency and physical-activity questions therefore produce rather rough estimates of dietary behaviour and physical activity. As a result, it was decided to use the information obtained to rank individuals – rather than the continuous outcomes – as the outcomes for analysis. Studies which investigate the validity of self-reported food-frequency information showed that questionnaires underestimate the energy intake of individuals compared to 24-hour dietary recall or the doubly labelled water method [39], [40], [41]. Social desirability bias, as well as cognitive problems relating to recalling the frequency and amount of food and drinks consumed, seems to compromise the internal validity of food-frequency questionnaires. Reporting bias is particularly problematic if it differs systematically according to specific characteristics of the respondents, causing differential misclassification bias [41], [42]; this possibility cannot be completely excluded in this study.

### Conclusions

In Germany, adults with a lower level of education consume energy-dense food more frequently than adults with a higher education level, although they consume alcohol less frequently. Higher levels of physical work activity among adults with lower education may partly explain why they consume more sugar-rich food. Thus, social disparities in sugar-rich food intake seem not only to be attributable to differences in knowledge, attitudes and personal preference; they also seem to be structurally determined, since physical work activity is a structural factor. Educational disparities in sugar- and fat-rich food intake observed in the total population seem to result mainly from strong educational disparities in sugar- and fat-rich food intake among persons with high energy expenditure. Health-promotion interventions aimed at improving healthy dietary behaviour should focus on physical workers with high levels of total energy expenditure (mainly lower-educated) and help them develop strategies to compensate their high demand for energy by consuming ‘healthy’, high-quality, energy-dense foods that are rich in complex carbohydrates and plant fats, and contain low amounts of sugar and saturated fats.
